# Chemoenzymatic Cascade Synthesis of Optically Pure Alkanoic Acids by Using Engineered Arylmalonate Decarboxylase Variants

**DOI:** 10.1002/chem.201806339

**Published:** 2019-03-12

**Authors:** Junichi Enoki, Carolin Mügge, Dirk Tischler, Kenji Miyamoto, Robert Kourist

**Affiliations:** ^1^ Junior Research Group for Microbial Biotechnology Ruhr-University Bochum Universitätstraße 150 44780 Bochum Germany; ^2^ Department of Biosciences and Informatics Keio University 3-14-1 Hiyoshi Kohoku-ku 22308522 Yokohama Japan; ^3^ Institute of Molecular Biotechnology Graz University of Technology Petersgasse 14 8010 Graz Austria

**Keywords:** arylmalonate decarboxylase, biocatalysis, cascade reactions, enzymes, hydrogenation

## Abstract

Arylmalonate decarboxylase (AMDase) catalyzes the cofactor‐free asymmetric decarboxylation of prochiral arylmalonic acids and produces the corresponding monoacids with rigorous *R* selectivity. Alteration of catalytic cysteine residues and of the hydrophobic environment in the active site by protein engineering has previously resulted in the generation of variants with opposite enantioselectivity and improved catalytic performance. The substrate spectrum of AMDase allows it to catalyze the asymmetric decarboxylation of small methylvinylmalonic acid derivatives, implying the possibility to produce short‐chain 2‐methylalkanoic acids with high optical purity after reduction of the nonactivated C=C double bond. Use of diimide as the reductant proved to be a simple strategy to avoid racemization of the stereocenter during reduction. The developed chemoenzymatic sequential cascade with use of *R*‐ and *S*‐selective AMDase variants produced optically pure short‐chain 2‐methylalkanoic acids in moderate to full conversion and gave both enantiomers in excellent enantiopurity (up to 83 % isolated yield and 98 % *ee*).

## Introduction

Enantiopure 2‐methyl‐substituted carboxylic acids are widely used as active pharmaceutical ingredients, building blocks, and fragrance and aroma compounds.^1^, ^2^ For instance, 2‐methylbutanoic acid derivatives are present in a wide range of fermented products such as bread, cheese, and several alcoholic beverages, contributing to their complex flavor.^3^ (*S*)‐2‐methylbutanoic acid is a precursor for the synthesis of the cholesterol‐lowering drug pravastatin.^4^ (*R*)‐2‐Methylbutanoic acid is part of the sex pheromone of the invasive species *Acutaspis albopicta*.^5^ (*S*)‐2‐Methylhexanoic acid is a constituent of the cytotoxic marine natural compound palau′imide.^6^ An efficient access to both pure enantiomers of short‐chain 2‐methyl‐substituted alkanoic acids would therefore pose a significant contribution to the development of numerous fine chemicals. Although a wide range of biocatalytic methods for the synthesis of arylaliphatic 2‐substituted carboxylic acids have been developed, including esterases, dehydrogenases, amidases, and decarboxylases,^1^ the synthesis of small chiral aliphatic carboxylic acids is challenging because of the difficulty to discriminate between two structurally very similar substituents. Lipase‐catalyzed kinetic resolution of 2‐methylalkanoic acids achieved moderate optical purities, but is inherently limited to 50 % maximum yield.^7^ Asymmetric hydrogenation over a heterogeneous Pd^0^ catalyst in the presence of cinchona derivatives as the chiral ligands led to moderate enantioselectivities.^8^ By using BINAP and Ru^II^, a series of tiglic acid derivatives was reduced with optical purities ranging from 79 to 97 % *ee*.^9^ These recent achievements underline the difficulty to prepare these challenging compounds.

**Figure 1:**
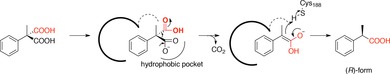
Reaction mechanism of wild‐type AMDase. The pro‐*S* carboxyl residue is marked in red. Dotted and bold lines represent the alkyl‐ and aryl‐binding pocket, respectively.

Arylmalonate decarboxylase from *Bordetella bronchiseptica* (AMDase) catalyzes the cofactor‐free asymmetric decarboxylation of prochiral 2‐methyl‐2‐arylmalonic acids with outstanding enantioselectivity, producing optically pure (*R*)‐2‐arylpropionates^10^ in a four‐step reaction (Figure 1).^11^, ^12^, ^13^, ^14^, ^15^, ^16^, ^17^ First, the pro‐*S* carboxyl residue of the substrate forms multiple hydrogen bonds with polar residues in an oxyanion hole. In this step the size of the two substituents in α position determines their accommodation in the so‐called alkyl‐ or aryl‐binding pockets of the active site. Second, the pro‐*R* carboxyl group is selectively decarboxylated by ground state destabilization in a hydrophobic pocket. Third, the generated enolate intermediate is stabilized by the oxyanion hole and the delocalized π‐electron system within the substrate. Finally, the catalytic cysteine residue transfers a proton to the planar intermediate in a stereospecific manner.

Several AMDase variants have been generated by protein engineering to alter the enzymatic performance such as the introduction of artificial racemase activity,^18^ complete inversion of enantioselectivity,^19^ and activity improvement of both *R*‐ and *S*‐selective AMDase variants.^20^, ^21^, ^22^ Owing to these investigations, AMDase decarboxylation is now applicable for the asymmetric synthesis of both enantiomers of 2‐substituted propionates with high optical purity. Okrasa et al. reported that wild‐type AMDase catalyzes the asymmetric decarboxylation of methylvinylmalonic acids with outstanding *R* enantioselectivity.^15^ This substrate specificity of AMDase suggests the potential applicability of AMDase and its variants to produce both enantiomers of short‐chain 2‐methylalkanoic acids. Considering that AMDase does not accept substrates without a delocalized π‐electron system,^10^, ^15^ we envisioned a cascade by combining the enzymatic synthesis of intermediary 2‐methylalk‐3‐enoic acids followed by a chemical C=C double bond reduction.

The development of chemoenzymatic cascade reactions, that is, one‐pot consecutive multiple reactions combining enzymatic reactions with chemical catalysis, has recently received increasing attention.^23^, ^24^, ^25^ This reaction concept allows to combine the strengths of biological and chemical catalysts in a one‐pot reaction system, save downstream work‐up processes, and addresses challenges such as the instability of reaction intermediates. Especially, various catalytic reactions with use of transition‐metal catalysts have been successfully combined with enzymatic reactions under aqueous or nonaqueous conditions.^26^, ^27^, ^28^, ^29^ The chemocatalytic reactions incorporated in such chemoenzymatic cascade systems so far, such as cross coupling^26^ and metathesis,^29^ greatly expand the catalytic scope of biocatalysis. Indeed, the hydrogenation of nonactivated C=C double bonds is extremely difficult for biocatalysis: For example, ene‐reductases catalyze an asymmetric hydrogen transfer towards activated 2,3‐unsaturated compounds, but are inactive towards nonactivated alkenes.^30^ The gut bacterium *Lactobacillus plantarum* possesses a polyunsaturated fatty acid metabolism pathway, in which a single nonactivated double bond of linoleic acid is reduced by using four different enzymes in six reaction steps.^31^ On the contrary, the hydrogenation of nonpolarized C=C double bonds is frequently performed with use of transition‐metal catalysts.

**Scheme 1:**
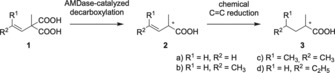
Chemoenzymatic one‐pot two‐step reaction for the synthesis of optically pure 2‐methylalkanoic acids **3**.

Herein, we report a chemoenzymatic cascade reaction to produce optically pure short‐chain 2‐methylalkanoic acids by combining the enzymatic asymmetric decarboxylation of methylvinylmalonic acid derivatives **1** and the chemical reduction of the nonactivated C=C double bond (Scheme 1).

## Results and Discussion

For the construction of this cascade system, several facts needed to be considered. First, although the activity of AMDase wild‐type (AMD‐WT) had already been reported in the synthesis of (*R*)‐**2**, the catalytic performance of other AMDase variants remained to be confirmed. Especially the mutations in the *S*‐selective AMDase variants with a shifted catalytic cysteine residue (G74C/C188G) were expected to significantly affect substrate recognition in the active site. Second, the chemical reduction should not show any side reactivity. It is known that transition‐metal catalysts can cause double bond migration to form the α,β‐unsaturated compound,^32^ which may lead to a partial racemization of the optically pure intermediates **2**. Third, the reaction conditions should be compatible with both reaction steps. Therefore, a potential inhibition of the chemical reduction had to be carefully evaluated in this designed cascade.

**Figure 2:**
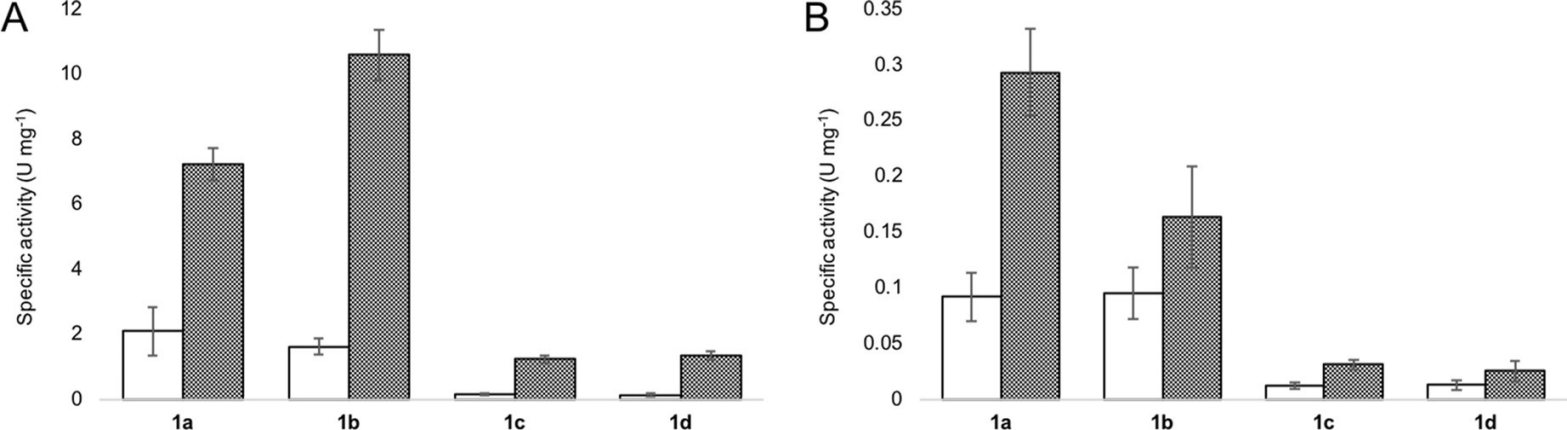
Specific activity of four AMDase variants towards substrates **1**. A) *R*‐Selective AMD‐WT (blank) and AMD‐IPLL (filled). B) *S*‐Selective AMD‐CLG (blank) and AMD‐CLGIPL (filled). U=μmol min^−1^.

To evaluate the effect of mutations within the hydrophobic pocket (V43I/A125P/V156L/M159L) and/or the catalytic cysteine residue (G74C/C188G), the specific activities of four AMDase variants, two being *R*‐selective (AMD‐WT and V43I/A125P/V156L/M159L, AMD‐IPLL^22^) and two being *S*‐selective (G74C/M159L/C188G, AMD‐CLG,^20^ and V43I/G74C/A125P/V156L/M159L/C188G, AMD‐CLGIPL^21^), towards the substrates **1** were determined (Figure 2 and Table S1). AMD‐CLGIPL (with mutations on the hydrophobic pocket and the catalytic cysteine residue) showed a 25‐ to 65‐fold activity decrease compared to AMD‐IPLL (with mutations on the hydrophobic pocket). This fact suggests that the enantioselectivity‐inverting shift of the cysteine residue in the active site critically disrupts the catalytic performance of AMDase towards the alkenyl substrates **1**. Nevertheless, both *R*‐ and *S*‐selective variants with mutations in the hydrophobic pocket, AMD‐IPLL and AMD‐CLGIPL, showed 3.5–10, respectively 1.7–3.2 times higher activity than their corresponding prior generations AMD‐WT and AMD‐CLG. The hydrophobic pocket causes ground‐state decarboxylation of the pro‐*R* carboxyl residue of the substrates **1** (Figure 1). This implies that a structural optimization of the hydrophobic environment, leading to enhanced conversion rates for arylmalonic acids,^20^, ^21^, ^22^ also positively affects substrate decarboxylation. It should be noted that the activity of AMD‐IPLL and AMD‐CLGIPL towards a typical arylmalonic acid derivative (up to 209 and 55 U mg^−1^, respectively)^22^ is 19–170 and 190–2200 times higher, respectively, than that towards the here‐discussed alkenyl substrates **1**. This may in part reflect the lower capacity of the single C=C double bond to stabilize the enolate intermediate during the transition state compared to that of an aromatic system.

Next, the enantioselectivity of the improved variants AMD‐IPLL and AMD‐CLGIPL was evaluated. Enzymatic reactions with use of crude cell extract including either of the AMDase variants showed full conversion of **1** at a concentration of 10 mm and generated the monoacids **2** with high optical purities with one exception (Table 1): *S*‐selective AMD‐CLGIPL produced (*S*)‐**2 a** with only 66 % *ee* (*S*). The same value was obtained from experiments with purified enzyme, ruling out any racemizing side reactions from the cellular debris. In control reactions, neither significant spontaneous decarboxylation in reactions without enzyme nor significant racemization of product **2** during prolonged incubations in the presence of crude cell extract were observed.

**Table 1:** Conversion and enantioselectivity of AMDase variants towards methylvinylmalonic acid derivatives **1**.^[a]^

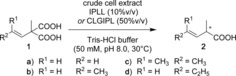					
Entry	Substrate	Variant	Time [h]	Conversion [%]	*ee* [%]
1	**1 a**	IPLL	1	>99	97 (*R*)
2	**1 a**	CLGIPL	3	>99	66 (*S*)
3	**1 b**	IPLL	1	>99	>99 (*R*)
4	**1 b**	CLGIPL	3	98	98 (*S*)
5	**1 c**	IPLL	3	90	>99 (*R*)
6	**1 c**	CLGIPL	3	85	>99 (*S*)
7	**1 d**	IPLL	3	98	>99 (*R*)
8	**1 d**	CLGIPL	18	>99	>99 (*S*)

[a] Reaction conditions: Tris‐HCl buffer (50 mm, pH 8.0 at 30 °C), substrate **1** (10 mm), 10 % (*v*/*v*) or 50 % (*v*/*v*) of crude cell extract including IPLL or CLGIPL, respectively. Reaction temperature: 30 °C.

Whilst (*R*)‐**2 a** is produced with high optical purity by both, the *R*‐selective AMD‐WT^15^ and AMD‐IPLL (entry 1 in Table 1), the stereo‐determining double mutation G74C/C188G in variant AMD‐CLGIPL can alter the enzyme–ligand binding. This may hypothetically result in a reduced discrimination between the methyl and vinyl substituents of the substrates within the mutated active site, eventually leading to a marked decrease of enantioselectivity for the small substrate **1 a**. Other possible explanations for this unexpected result may be rearrangement processes of the ligand in the transition state or reprotonation by an alternate, unwanted, proton donor such as water.

Having confirmed the possibility to produce enantiomerically enriched **2**, we then investigated the subsequent reduction. The nonstereoselective reduction of nonpolarized C=C double bonds is typically accomplished with heterogenous catalysts such as Pd^0^, Rh^0^, Raney nickel, or PtO_2_ under a H_2_ atmosphere.^33^ For the reduction of optically pure **2**, the tendency of transition metals to promote isomerization reactions^32^ poses a risk. After complete decarboxylation of **1 d** (10 mm) catalyzed by crude cell extract containing AMDase IPLL, addition of Pd/C and H_2_ resulted in complete reduction of the intermediary (*R*)‐**2 d** within 1.5 h. The resulting (*R*)‐**3 d** could be isolated in 83 % yield, showing that the combination of the reducing catalyst with crude cell extracts is straightforward. As expected, however, the Pd^0^ catalyst induced partial isomerization of the stereocenter: While (*R*)‐**2 d** was formed as pure enantiomer (>99 % *ee*), the final product (*R*)‐**3 d** had a significantly reduced optical purity (81 % *ee*).

As an alternative C=C double bond reduction approach, we investigated the in situ generation of diimide. Diimide is a hydrogen donor that can selectively reduce nonpolarized unsaturated bonds by a concerted hydrogen transfer without isomerization.^34^ Diimide itself is unstable and has to be generated in situ from its precursor hydrazine by using oxidation catalysts. Several catalysts have been reported for the in situ generation of diimide,^35^, ^36^, ^37^, ^38^ and those reactions were performed in organic solvent in most cases. To circumvent extra work‐up processes, a water‐soluble Cu^II^ catalyst,^39^ CuCl_2_, was selected for hydrazine oxidation. The reduction activity was confirmed by using a model substrate, *trans*‐3‐hexenoic acid **4**, under aqueous conditions (Table 2, entry 4).

**Table 2:** Catalyst screening for in situ diimide formation.^[a]^

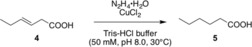				
Entry	Cat. concn [equiv]	N_2_H_2_ concn [equiv]	Time [h]	Conversion [%]
1	0.001	10	5	38
2	0.001	20	5	44
3	0.001	20	17	92
4	0.01	20	17	99

[a] Reaction conditions: Tris‐HCl buffer (50 mm, pH 8.0 at 30 °C), substrate **4** (10 mm) under aerobic conditions. Reaction temperature: 30 °C.

The sequential chemoenzymatic cascade reaction was then performed by combining AMDase decarboxylation and C=C double bond reduction through the in situ generated diimide by using a copper salt. GC‐FID analysis of the final product **3** presented moderate to full conversion over two steps and, more importantly, a conserved high enantiomeric excess (Table 3). This result implies that the reduction activity of diimide was not influenced by contaminants from *E. coli* cell free extract and that racemizing or inhibiting side reactions could be circumvented with this approach. Interestingly, the lowest reduction rate was observed for the production of (*R*)‐ and (*S*)‐**3 c** (Table 3, entries 5 and 6). Diimide selectively reduces terminal olefins and is less reactive towards internal and especially multisubstituted double bonds.^38^ With use of **1 a** on a milligram‐scale (110 mg, 0.76 mmol), the reaction proceeded to complete conversion over both steps and allowed the isolation of (*R*)‐**3 a** with excellent optical purity (98 % *ee*) and 83 % yield (64 mg, 0.63 mmol).

**Table 3:** Sequential chemoenzymatic cascade combining AMDase decarboxylation and in situ generation of diimide.^[a]^

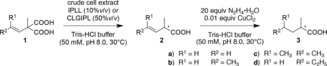				
Entry	Substrate	Time [h]^[b]^	Conversion [%]	*ee* of **3** [%]
1	**1 a**	23	>99	98 (*R*)
2	**1 a**	23	>99	66 (*S*)
3	**1 b**	23	80	>99 (*R*)
4	**1 b**	23	78	>99 (*S*)
5	**1 c**	23	20	>99 (*R*)
6	**1 c**	23	11	>99 (*S*)
7	**1 d**	23	86	>99 (*R*)
8	**1 d**	23	89	>99 (*S*)

[a] After completion of the crude cell extract biocatalysis (see Table 1), hydrazine monohydrate (20 equiv) and CuCl_2_ (0.01 equiv) were added to the reaction system. Reaction temperature: 30 °C. [b] Reaction times for C=C reduction step.

## Conclusion

The feasibility to combine enzymatic decarboxylation and chemical reduction was demonstrated in this study. The here developed chemoenzymatic reaction cascade can be operated in sequential manner to give access to optically pure short‐chain 2‐methylalkanoic acids **3**.

Our work presents a potential synthetic applicability of AMDase biocatalysis in combination with metallo‐ and organo‐chemical reactions in the same reaction system, but also points out difficulties with side reactivities and selectivity issues in the combination of the different catalysts. The hydrogenation of nonactivated C=C double bonds by using a heterogeneous Pd^0^ catalyst initially resulted in significantly decreased optical purity of the final products **3**, which is most probably due to partial isomerization. An alternative reduction approach by applying in situ generated diimide as the reductant produced the final products **3** in moderate to full conversion and with conserved high enantiomeric excess. While partial racemization could be overcome by the choice of an appropriate reduction approach, the reduced selectivity of *S*‐selective AMDase variants towards the shortest alkenyl substrate **1 a** may be addressed in future work.

## Experimental Section

### General

Substrates **1** were synthesized as described in the Supporting Information. All other chemicals used in this study were purchased from commercial suppliers. A Bruker (Rheinstetten, Germany) DPX‐200 NMR device was used for ^1^H and ^13^C NMR measurements. High performance liquid chromatography (HPLC) analyses were performed with an AZURA HPLC System (Knauer, Berlin, Germany) with a NUCLEODUR C18 Pyramid column (5 μm; 4.6×250 mm; Macherey–Nagel, Düren, Germany). The enantiomeric excess of intermediates **2** and products **3** was determined by chiral gas chromatography with a flame ionization detector (GC‐FID) by using a Shimazu GC Plus 2010 device (Shimazu, Kyoto, Japan) with a chiral column FS‐Hydrodex‐β ‐6TBDM (Macherey–Nagel, Düren, Germany).

The codon‐optimized genes of *B. bronchiseptica* AMD‐WT (Un*i*ProtKB/Swiss‐Prot: Q05 115, PDB: 3DG9) and its variants V43I/A125P/V156L/M159L (AMD‐IPLL), G74C/M159L/C188G (AMD‐CLG), and V43I/G74C/A125P/V156L/M159L/C188G (AMD‐CLGIPL) were cloned into a pET28a vector with treatment of restriction enzymes NdeI, and XhoI. *E. coli* BL21(DE3) competent cells (Agilent Technologies, Santa Clara, CA, USA) were used for protein expression.^22^

### Expression and purification of recombinant AMDase variants

Expression of AMDase variants was performed with *E. coli* BL21(DE3) cells harboring plasmid DNA pET28a encoding the genes of AMDase wild‐type or its variants in LB medium (50 mL) with kanamycin (30 μg mL^−1^) at 37 °C. After the OD600 reached 0.5, overexpression was induced by addition of IPTG (1 mm) and the cells cultivated overnight at 30 °C. The cells were harvested by centrifugation (6000×g, 20 min, 4 °C) and stored at −20 °C. AMDase variants for biocatalyses were either used as crude cell extracts or purified by Ni‐affinity column chromatography following the manufacturer's instructions (HisPurTM Ni‐NTA Resin, Thermo Fisher Scientific, MA, USA). The buffer of the isolated elution fraction was replaced with Tris‐HCl buffer (50 mm, pH 8.0, 30 °C) by using centrifugal filter units (Vivaspin® Turbo15, Sartorius, Göttingen, Germany). The protein concentration was determined by UV absorption with use of NanoDrop (Thermo Fisher Scientific, MA, USA). The theoretical extinction coefficient at 280 nm and molecular weight of AMDase variants are 13 200 cm^−1^ m ^−1^ and 25.8 kDa, respectively.

### Determination of enzymatic activity

Purified AMDase wild‐type and its variants were used for specific activity measurement towards alkenyl substrates **1**. The enzymatic reaction was performed under the following conditions: substrate **1** (10 mm), Tris‐HCl (50 mm, pH 8.0 at 30 °C), wild‐type (0.05–0.2 mg mL^−1^ ), IPLL (0.025–0.1 mg mL^−1^), CLG (0.75–2 mg mL^−1^), and CLGIPL (0.5–1 mg mL^−1^) of AMDase variants at reaction temperature of 30 °C. The reaction was quenched by adding a mixture of acetonitrile, 2 m HCl, and 2‐phenylacetic acid or 2‐phenylpropionic acid (10 mm) as an internal standard (7:2:1) to reach 50 % of the final sample volume. The consumption of starting materials **1** was quantified by using HPLC at a detection wavelength of 200 nm and 1.0 mL min^−1^ isocratic mobile phase (acetonitrile/dH_2_O/trifluoroacetic acid=50:50:0.1) with a column temperature of 25 °C. The retention times of substrates **1** and products **2** were 2.88 (**1 a**), 3.80 (**2 a**), 3.13 (**1 b**), 4.45 (**2 b**), 3.36 (**1 c**), 5.31 (**2 c**), 3.60 (**1 d**), and 5.73 min (**2 d**).

### Analytical scale chemoenzymatic one‐pot two‐step combination of enzymatic decarboxylation and C=C reduction

The first‐step decarboxylation was performed on 0.5 mL scale with AMDase variants IPLL and CLGIPL. The reaction conditions were: substrate **1** (10 mm), Tris‐HCl (50 mm, pH 8.0 at 30 °C), 10 % *v*/*v* (IPLL) or 50 % *v*/*v* (CLGIPL) of the crude extract of AMDase variants (ca. 75 mg wetted cell pellet mL^−1^) at 30 °C. Substrate conversion was followed by HPLC analysis as described above. After full conversion of the substrates **1**, the second‐step C=C reduction was performed by addition of hydrazine monohydrate (20 equiv) and CuCl_2_ (0.01 equiv). The reaction was quenched by the addition of 2 m HCl and subsequently the final products **3** were extracted with ethyl acetate. The combined organic phases were dried with anhydrous MgSO_4_. The conversion and enantiomeric excess of the final products **3** was analyzed by chiral GC‐FID. The retention times of the intermediates **2** and products **3** were 6.2 [(*R*)‐**2 a**], 6.0 [(*S*)‐**2 a**], 5.7 [(*R*)‐**3 a**], 5.5 [(*S*)‐**3 a**], 19.6 [(*R*)‐**2 c**], 20.4 [(*S*)‐**2 c**], 13.3 [(*R*)‐**3 c**], 13.5 [(*S*)‐**3 c**], 19.4 [(*R*)‐**2 d**], 18.4 [(*S*)‐**2 d**], 18.2 [(*R*)‐**3 d**], and 17.9 min [(*S*)‐**3 d**] with column temperature at 100 °C and 4.9 [(*R*)‐**2 b**], 4.5 [(*S*)‐**2 b**], 4.2 [(*R*)‐**3 b**], and 4.0 min [(*S*)‐**3 b**] at 120 °C.

**Caution**: Hydrazine is a cancer suspect agent.

### Preparative chemoenzymatic one‐pot two‐step reaction to produce 3 a

2‐Methyl‐2‐alkenylmalonate **1 a** (110 mg, 0.76 mmol) was dissolved in Tris‐HCl buffer (40 mL, 50 mm, pH 8.0 at 30 °C) containing 10 %v/v of the crude extract of an AMDase variant IPLL (ca. 75 mg wetted cell pellet mL^−1^). The biocatalysis was performed at 30 °C as stated above for 4 h and full conversion of substrate **1 a** was confirmed by TLC control. Next, hydrazine monohydrate (20 equiv) and CuCl_2_ (0.01 equiv) were added to the reaction solution and the mixture stirred at 30 °C under aerobic conditions. After the addition of 2 m HCl, the carboxylates were extracted with diethyl ether. The combined organic phases were dried with anhydrous MgSO_4_ and the solvent was evaporated. The final product **3 a** was isolated by flash column chromatography (diethyl ether/*n*‐pentane=1:1). Isolated yield: 83 % [64 mg, 0.63 mmol, 98 % *ee* (*R*)].

**2‐Methylbutanoic acid (3 a)**: ^1^H NMR (200 MHz, [D_6_]DMSO): *δ*=2.34–2.10 (m, 1 H), 1.69–1.18 (m, 2 H), 1.03 (d, *J=*6.9 Hz, 3 H), 0.84 ppm (t, *J=*7.4 Hz, 3 H); ^13^C NMR (50 MHz, [D_6_]DMSO): *δ*=177.3, 40.2, 26.2, 16.5, 11.4 ppm.

## Conflict of interest

The authors declare no conflict of interest.

## Supporting information

As a service to our authors and readers, this journal provides supporting information supplied by the authors. Such materials are peer reviewed and may be re‐organized for online delivery, but are not copy‐edited or typeset. Technical support issues arising from supporting information (other than missing files) should be addressed to the authors.

Supplementary
