# Supplementary material for: Chemoenzymatic Cascade Synthesis of Optically Pure Alkanoic Acids by Using Engineered Arylmalonate Decarboxylase Variants
Source: Chemistry. 2019 Mar 12;25(19):5071–6. doi: 10.1002/chem.201806339 (PMC6563808; doi:10.1002/chem.201806339)
Supplement: Supplementary file 1 — Supplementary [file CHEM-25-5071-s001.pdf]

CHEMISTRY
A European Journal

Supporting Information

Chemoenzymatic Cascade Synthesis of Optically Pure Alkanoic Acids by Using Engineered Arylmalonate Decarboxylase Variants

Junichi Enoki,[a] Carolin Mügge,[a] Dirk Tischler,[a] Kenji Miyamoto,[b] and Robert Kourist*[c]

chem_201806339_sm_miscellaneous_information.pdf

Author Contributions

J.E. Conceptualization: Equal; Investigation: Lead; Methodology: Lead; Writing – original draft: Lead; Writing – review & editing: Equal
C.M. Conceptualization: Equal; Formal analysis: Lead; Writing – original draft: Supporting; Writing – review & editing: Supporting
D.T. Writing – review & editing: Supporting
K.M. Conceptualization: Supporting; Writing – original draft: Supporting
R.K. Conceptualization: Lead; Data curation: Supporting; Formal analysis: Supporting; Investigation: Supporting; Writing – original draft: Lead; Writing – review & editing: Lead.

Contents

Experiments	S2
Table S1	S4
Compound Analytics: ^{1}H-NMR data	S5
Compound Analytics: ^{13}C-NMR data	S8
Compound Analytics: Chiral GC-FID chromatogram	S11
References	S13

Synthesis of 2-methyl-2-alkenylmalonic acids **1**

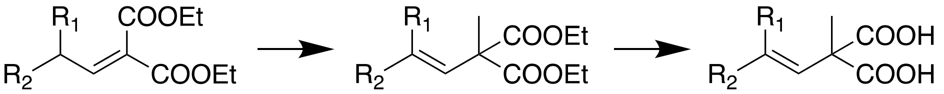

4 **5** **1**

a) R_1 = H, R_2 = H **c**) R_1 = CH_3, R_2 = CH_3
b) R_1 = H, R_2 = CH_3 **d**) R_1 = H, R_2 = C_2H_5

Diethyl ethylidenemalonate **4a** was purchased from a chemical supplier abcr (Karlsruhe, Germany). The other diethyl alkylidenemalonates **4b-d** were synthesized by standard Knoevenagel condensation reactions.

Diethyl 2-methyl-2-vinylmalonate **5a** was prepared according to a procedure described in literature.[1] Lithium diisopropyl amine (LDA) was generated by the addition of *n*-butyl lithium (1.6 M in hexane, 9.6 mmol) into anhydrous THF (20 mL) and diisopropylamine (11 mmol) at -78°C under N_2 atmosphere. After 10 min, *N*,*N'*-dimethylpropyleneurea (10 mL) and substrate **4a** (5.4 mmol) was added and the reaction solution was stirred for additional 45 min at -78°C. Iodomethane (8 mmol) was then applied and the reaction solution was stirred at room temperature for 18 hours. The reaction was quenched by addition of saturated NH_4Cl and the reaction mixture was extracted with ethyl acetate. The combined organic phase was dried over anhydrous $MgSO_4$, filtered and the solvents were evaporated. The product **5a** was purified with flash column chromatography (light petroleum : ethyl acetate = 40 : 1) to give a transparent oil (yield 19 %).

The other diethyl 2-methyl-2-alkenyl malonates **5b-d** were synthesized via methylation with sodium ethoxide as a base. Sodium ethoxide was generated by dropwise addition of ethanol (30 mL) to sodium (20 mmol) at 4°C with stirring for 1 hour. 10-13 mmol of **4b-d** were added, and after 30 min stirring on ice 1.3 mol-eq. iodomethane were applied for the reaction system. The reaction system was gradually warmed to room temperature and stirred for 18 hours. After the addition of saturated $NaHCO_3$, the reaction products were extracted by ethyl acetate. The combined organic phase was dried over anhydrous $MgSO_4$, filtered and solvents evaporated. The products **5b-d** were purified by flash column chromatography (light petroleum : ethyl acetate = 40 : 1) to give transparent oils in yields of 20-23 %.

2-methyl-2-alkenylmalonic acids **1** were synthesized according to literature procedure.[1,2] To a solution of substrates **5** (1-3.5 mmol) in 20 ml of ethanol, 10 ml of 8 M NaOH was added dropwise to the reaction system at 4°C. Afterwards, the reaction system was gradually warmed to room temperature and stirred for 18 hours. The reaction solution was neutralized by addition of 37 % HCl and ethanol was evaporated. After acidification with 2 M HCl, the carboxylates were extracted by ethyl acetate. The products **1** were crystallized as white solids using light petroleum and *tert*-butyl methyl ether (yield 72-86 %).

2-methyl-2-vinylmalonic acid (**1a**): ^1H NMR (200 MHz, dimethyl sulfoxide d6) δ 6.36-6.10 (m, 1H), 5.28-5.01 (m, 2H), 1.40 (s, 3H). ^{13}C NMR (50 MHz, dimethyl sulfoxide d6) δ 172.1, 137.3, 114.9, 55.5, 19.1.

2-methyl-2-(prop-1-enyl)malonic acid (**1b**): ^1H NMR (200 MHz, dimethylsulfoxide d6) δ 5.92-5.76 (m, 1H), 5.63-5.41 (m, 1H), 1.68 (dd, *J* = 6.4, 1.6 Hz, 3H), 1.36 (s, 3H). ^{13}C NMR (50 MHz, dimethyl sulfoxide d6) δ 172.5, 130.2, 124.9, 54.7, 19.8, 17.8.

2-methyl-2-(2'-methylprop-1'-enyl)malonic acid (**1c**): ^{1}H NMR (200 MHz, dimethylsulfoxide d6) δ 5.76-5.64 (m, 1H), 1.68 (s, 3H), 1.54 (s, 3H), 1.39 (s, 3H). ^{13}C NMR (50 MHz, dimethyl sulfoxide d6) δ 173.1, 133.8, 124.3, 53.3, 26.7, 22.6, 18.1.

2-methyl-2-(but-1-enyl)malonic acid (**1d**): ^{1}H NMR (200 MHz, dimethylsulfoxide d6) δ 5.98-5.78 (m, 1H), 5.68-5.47 (m, 1H), 2.16-1.96 (m, 2H), 1.39 (s, 3H), 0.97 (t, J = 7.4 Hz, 3H). ^{13}C NMR (50 MHz, dimethyl sulfoxide d6) δ 172.6, 131.6, 128.2, 54.6, 25.0, 19.9, 13.3.

Semi-preparative enzymatic decarboxylation of alkenyl substrates **1a-d**

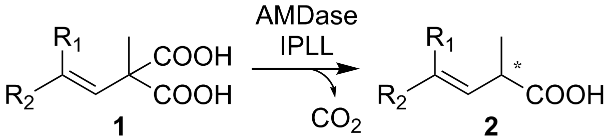

a) R_1 = H R_2 = H **c**) R_1 = CH_3 R_2 = CH_3
b) R_1 = H R_2 = CH_3 **d**) R_1 = H R_2 = C_2H_5

0.4 mmol of 2-methyl-2-alkenylmalonates **1** were dissolved in 40 ml of 50 mM Tris-HCl buffer (pH 8.0 at 30°C) containing 10 %v/v of the crude extract of AMDase variants (ca. 75 mg wetted cell pellet/mL). The biocatalysis was performed at 30°C. After full conversion of substrate **1** was confirmed by thin-layer chromatography (TLC) control, 2 M HCl was added to the reaction solution and the products were extracted by ethyl acetate. The combined organic phases were dried over anhydrous $MgSO_4$ and evaporated. The products **2** were isolated by flash column chromatography (light petroleum : ethyl acetate = 10:1) in 50-98 % yield.

2-methylbut-3-enoic acid (**2a**): ^{1}H NMR (200 MHz, acetone d6) δ 6.13-5.81 (m, 1H), 5.26-4.98 (m, 2H), 3.24-3.02 (m, 1H), 1.21 (d, J = 7.0 Hz, 3H). ^{13}C NMR (50 MHz, acetone d6) δ 175.4, 138.9, 116.0, 44.1, 17.1.

(*E*)-2-methylpent-3-enoic acid (**2b**): ^{1}H NMR (200 MHz, acetone d6) δ 5.71-5.45 (m, 2H), 3.18-2.95 (m, 1H), 1.68-1.60 (m, 3H), 1.21 (d, J = 7.0 Hz, 3H). ^{13}C NMR (50 MHz, acetone d6) δ 175.86, 131.6, 126.7, 43.2, 18.0, 17.8.

2,4-dimethylpent-3-enoic acid (**2c**): ^{1}H NMR (200 MHz, acetone d6) δ 5.22-5.07 (m, 1H), 3.41-3.20 (m, 1H), 1.72-1.63 (m, 6H), 1.14 (d, J = 7.0 Hz, 3H). ^{13}C NMR (50 MHz, acetone d6) δ 176.3, 133.9,125.4, 39.3, 25.8, 18.4, 18.0.

(*E*)-2-methylhex-3-enoic acid (**2d**): ^{1}H NMR (200 MHz, dimethylsulfoxide d6) δ 5.78-5.28 (m, 2H), 3.15-2.87 (m, 1H), 2.18-1.83 (m, 2H), 1.13 (d, J = 7.0 Hz, 3H), 0.95 (t, J = 7.4 Hz, 3H). ^{13}C NMR (50 MHz, dimethyl sulfoxide d6) δ 175.6, 132.4, 128.5, 41.9, 24.8, 17.2, 13.4.

Table S1. Specific activity of AMDase wild-type and its variants towards **1**

	Specific activity (U mg^{-1})*			
	Wild-type	IPLL	CLG	CLGIPL
**1a**	2.1 ± 0.74	7.2 ± 0.50	0.092 ± 0.022	0.29 ± 0.039
**1b**	1.6 ± 0.25	11 ± 0.78	0.095 ± 0.023	0.16 ± 0.045
**1c**	0.16 ± 0.033	1.2 ± 0.10	0.012 ± 0.0031	0.031 ± 0.0045
**1d**	0.13 ± 0.048	1.3 ± 0.12	0.013 ± 0.0045	0.025 ± 0.0089

*: U = µmol min^{-1}

Compound Analytics: ^{1}H-NMR data

1a

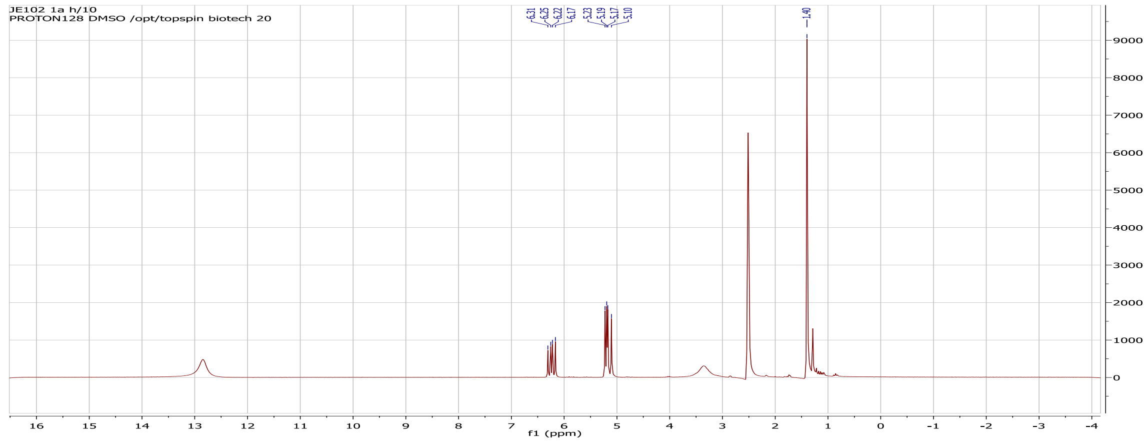

1b

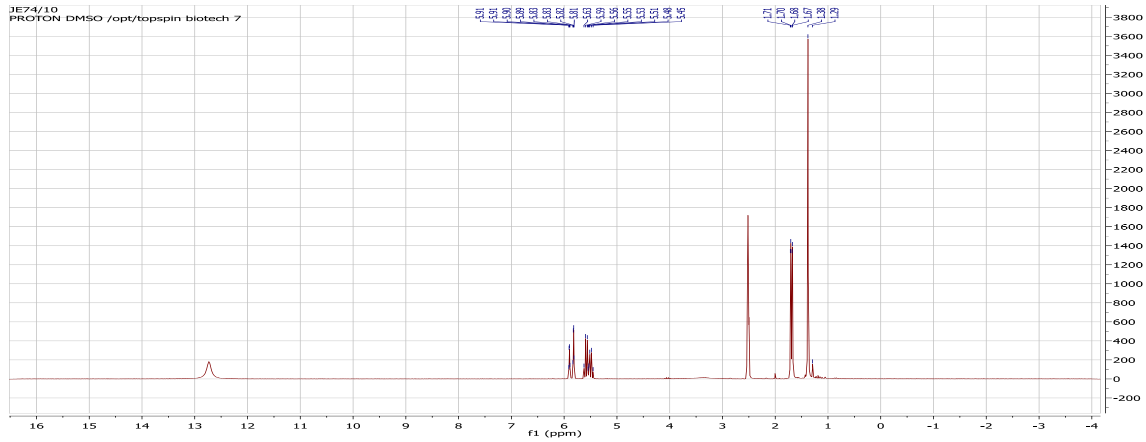

1c

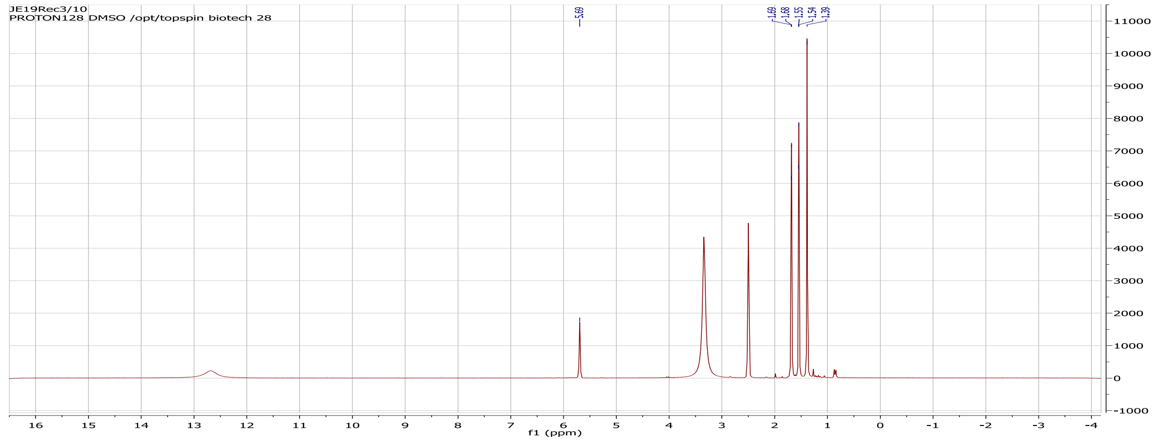

1d

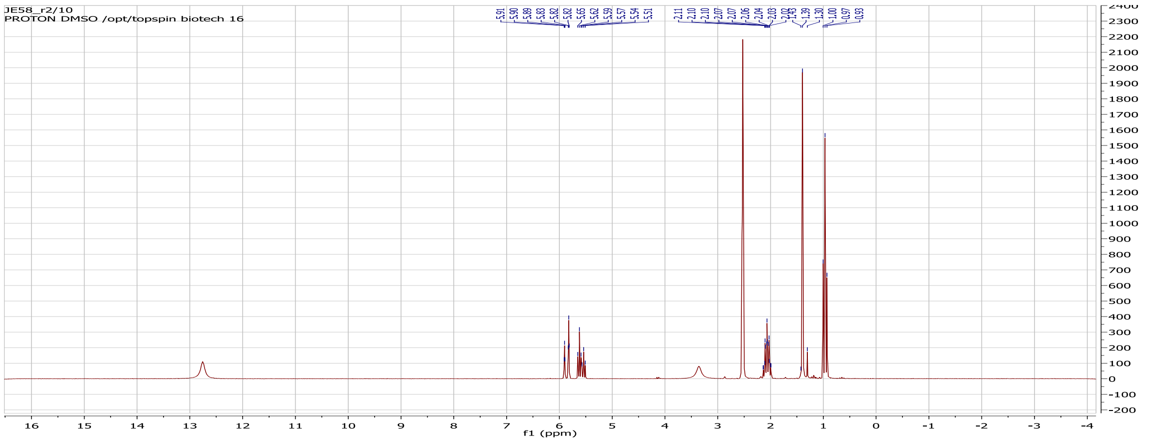
JE58_r2/10
PROTON DMSO /opt/topspin biotech 16
f1 (ppm)

2a

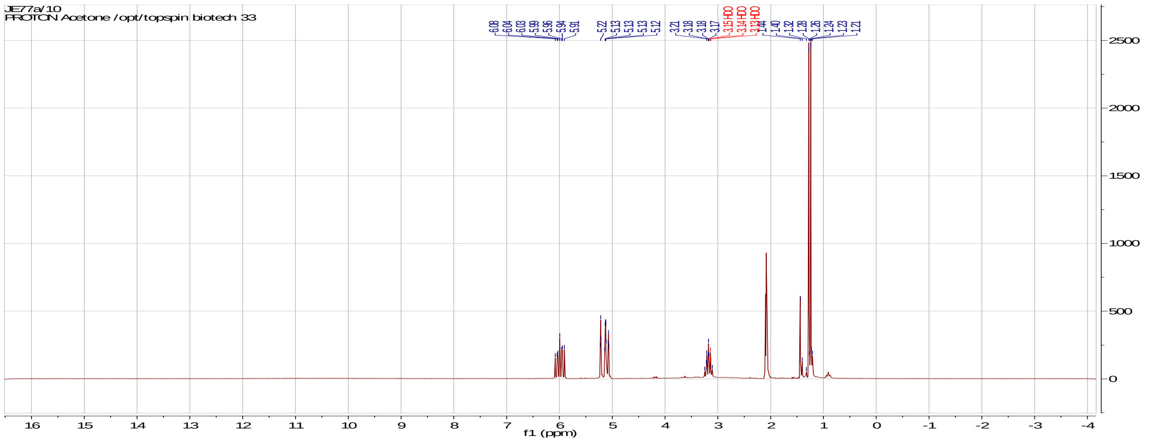
JE77a/10
PROTON Acetone /opt/topspin biotech 33
f1 (ppm)

2b

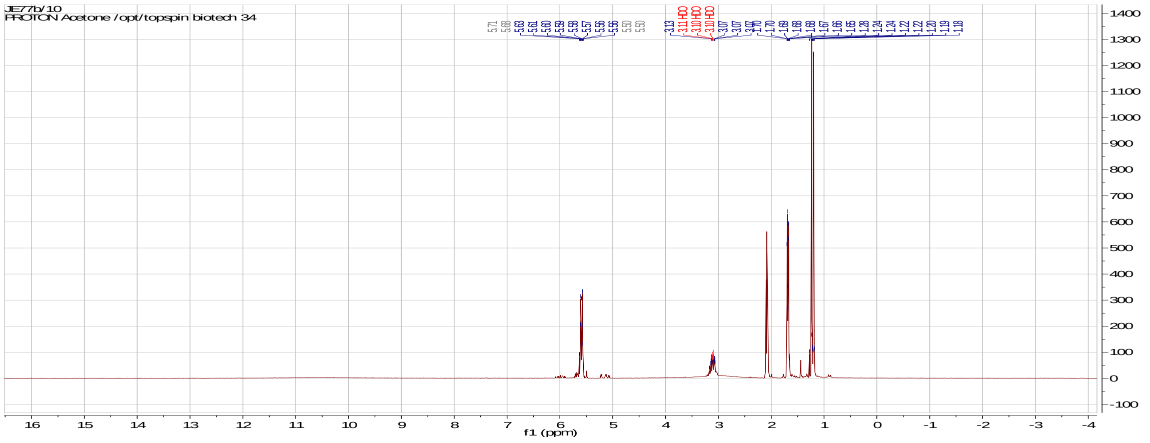
JE77b/10
PROTON Acetone /opt/topspin biotech 34
f1 (ppm)

2c

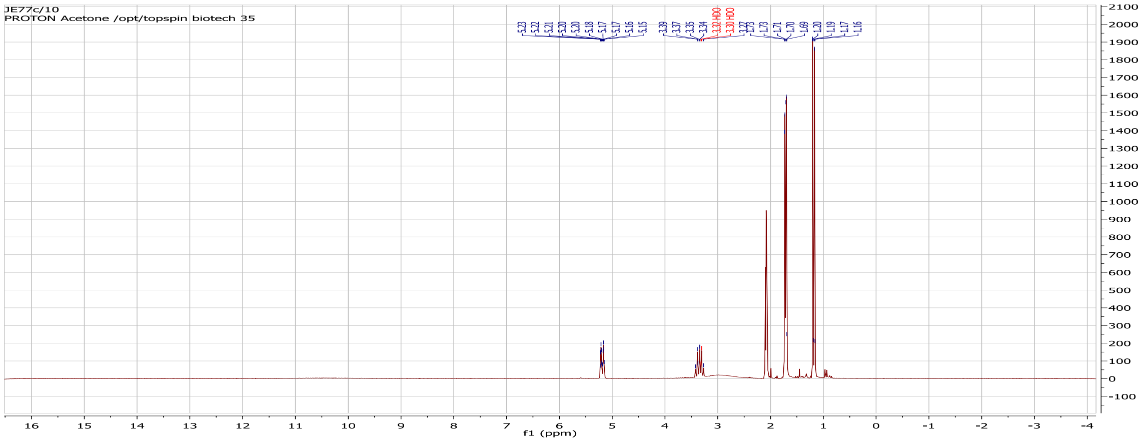
JE77c/10
PROTON Acetone /opt/topspin biotech 35
f1 (ppm)

2d

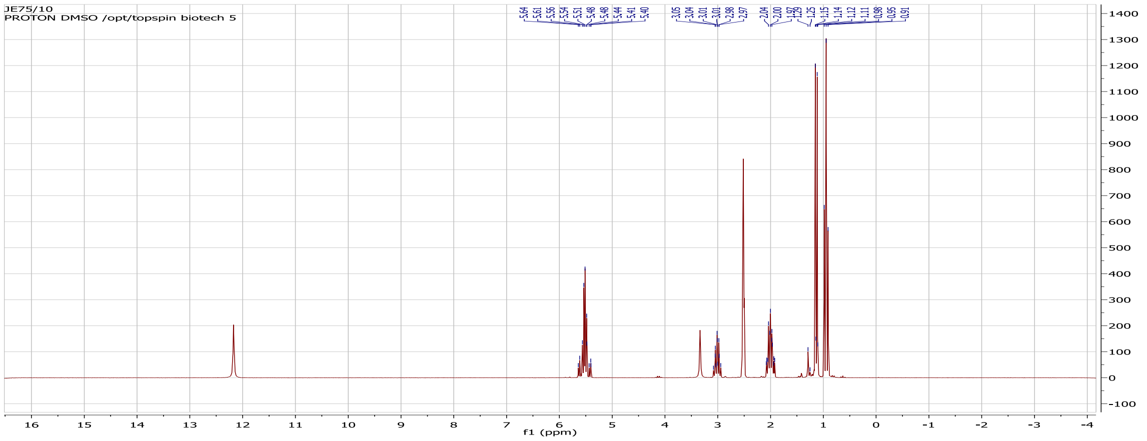
JE75/10
PROTON DMSO /opt/topspin biotech 5
f1 (ppm)

3a

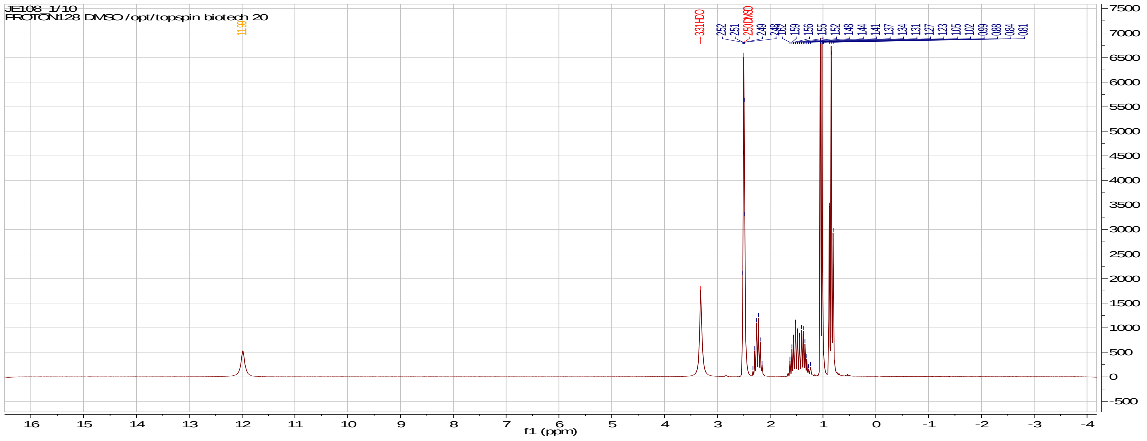
JE108_1/10
PROTON128 DMSO /opt/topspin biotech 20
f1 (ppm)

Compound Analytics: ^{13}C-NMR data

1a

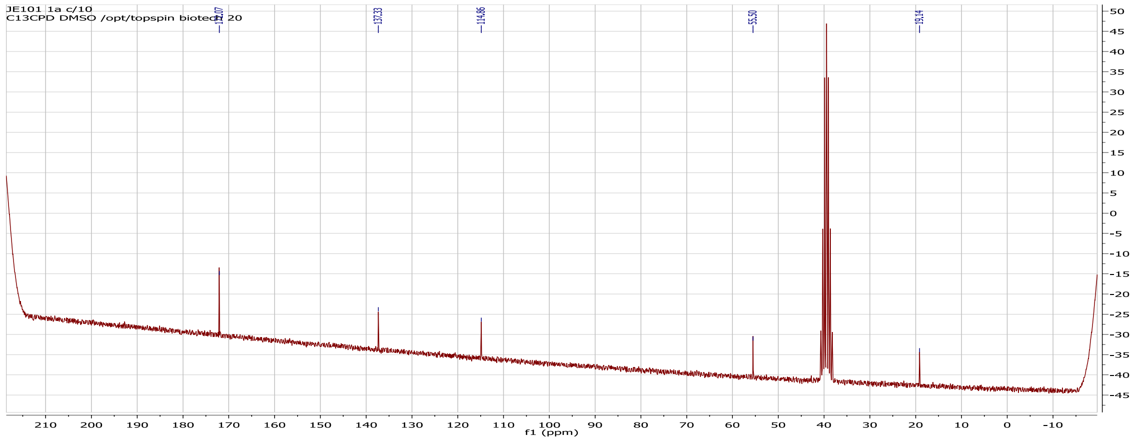

1b

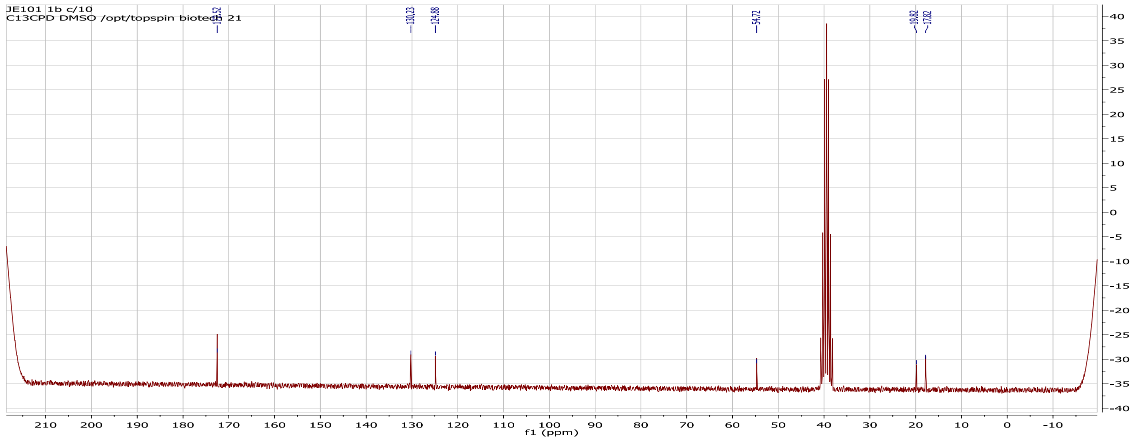

1c

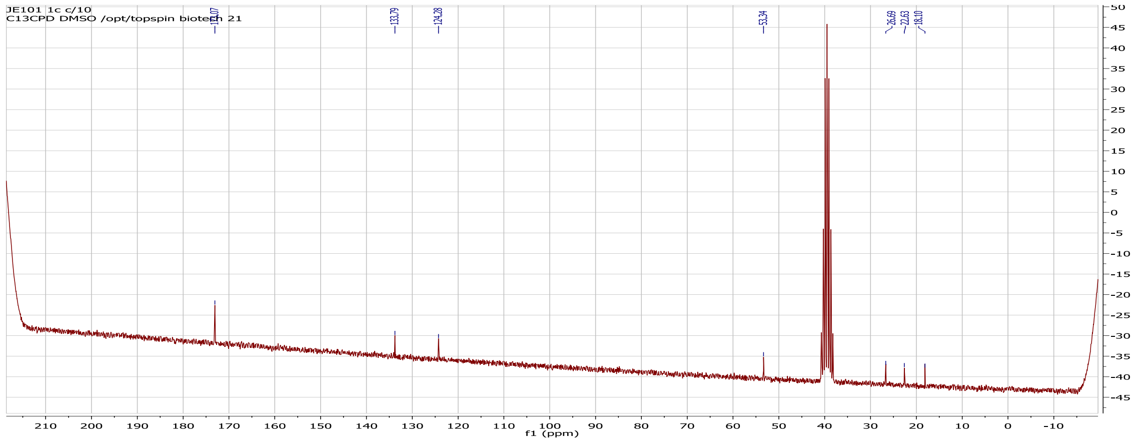

1d

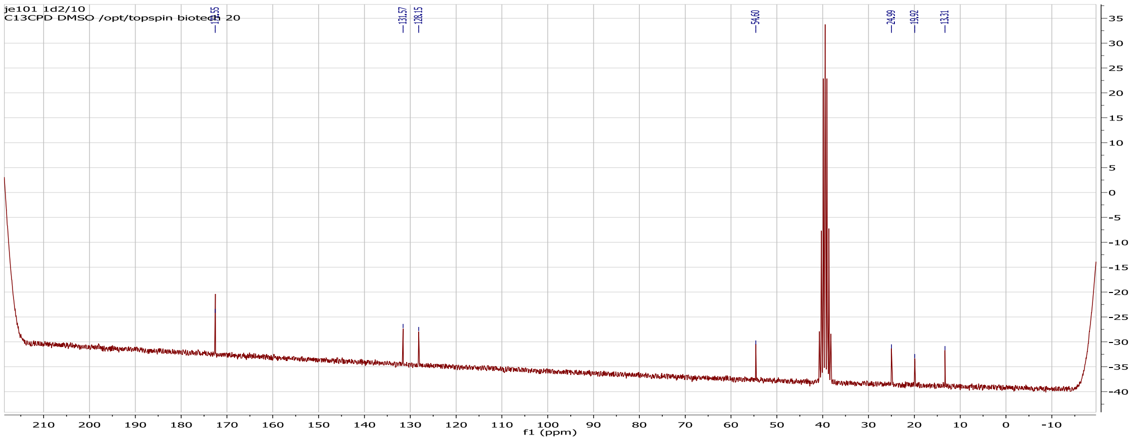

je101 1d2/10
C13CPD DMSO /opt/topspin biotech 20
131.57
128.15
54.60
24.99
19.92
13.31
f1 (ppm)

2a

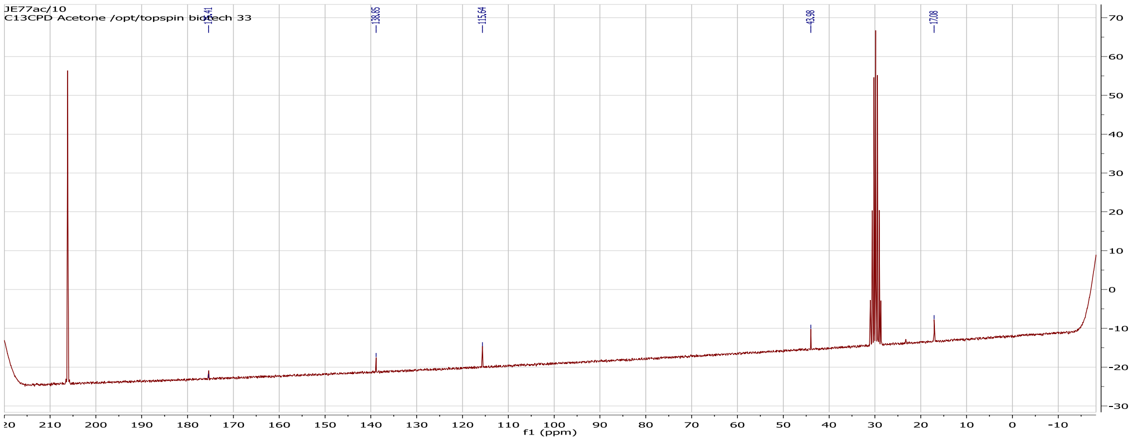

JE77ac/10
C13CPD Acetone /opt/topspin biotech 33
138.85
115.64
43.98
17.08
f1 (ppm)

2b

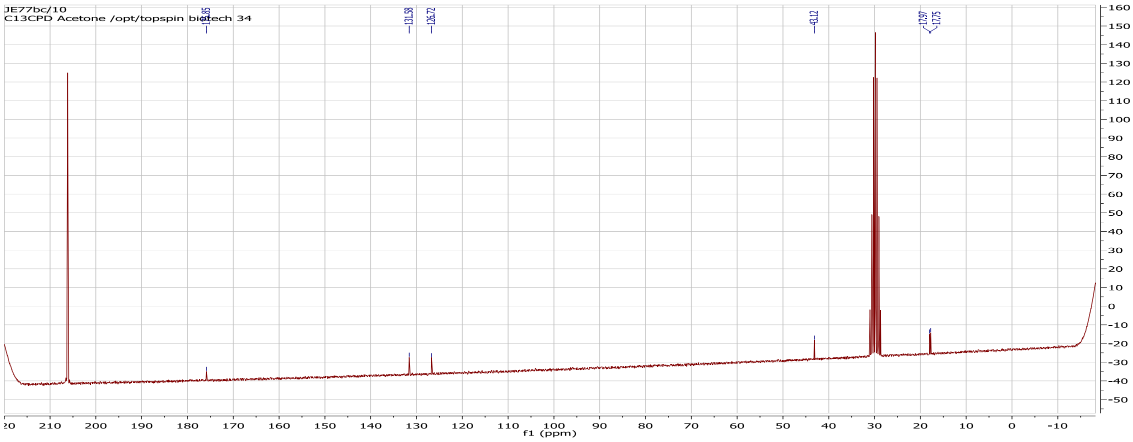

JE77bc/10
C13CPD Acetone /opt/topspin biotech 34
131.58
126.72
43.12
17.97
17.75
f1 (ppm)

2c

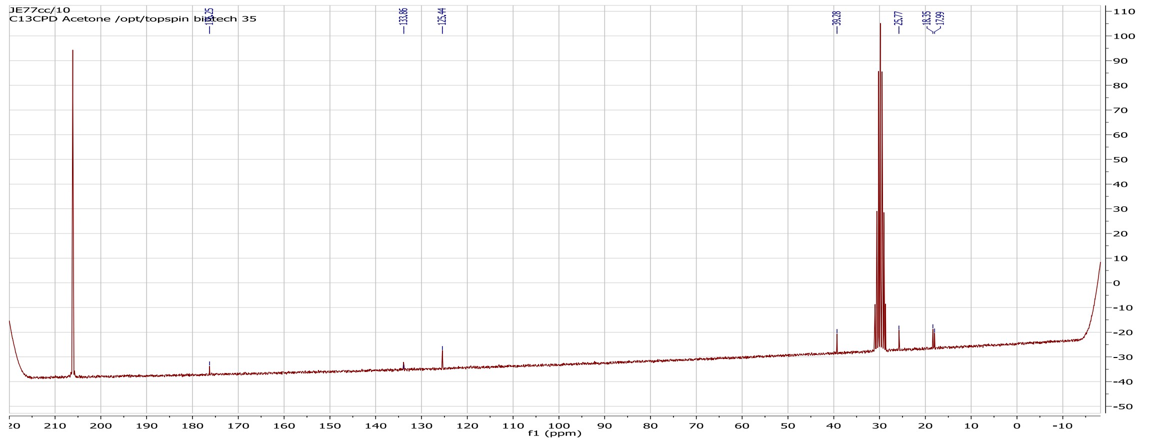

2d

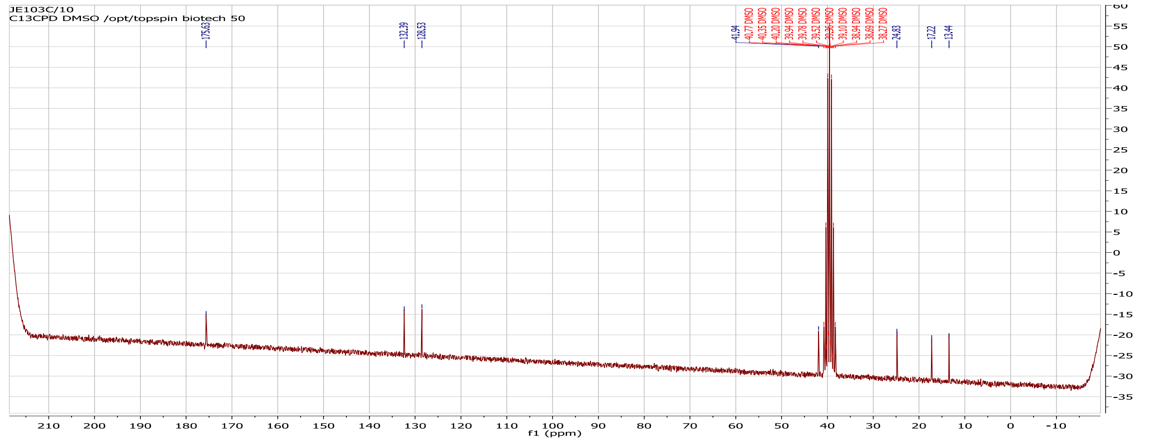

3a (The ^{13}C-NMR spectra was obtained from attached proton test (APT))

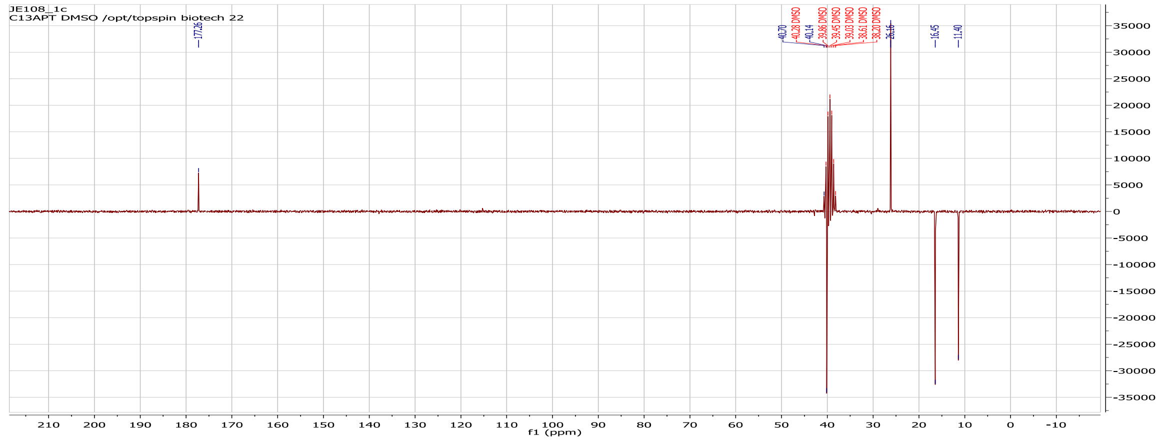

Compound analytics: Chiral GC-FID chromatogram

2a

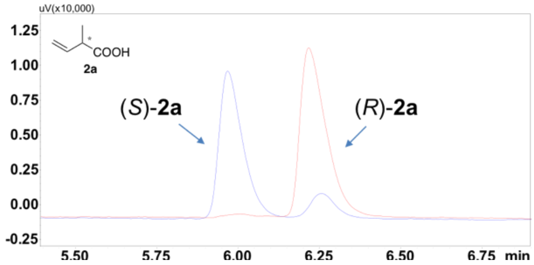

2b

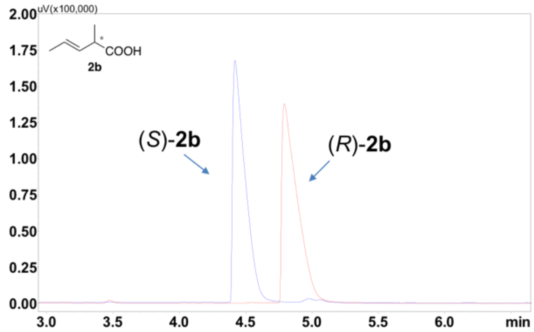

2c

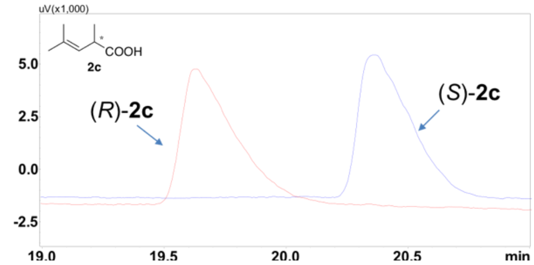

2d

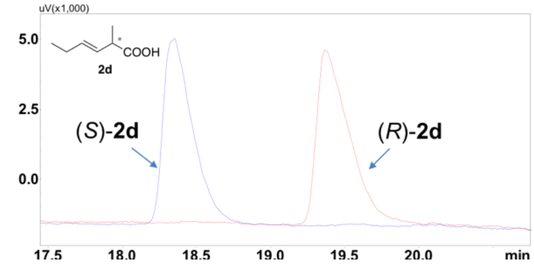

3a

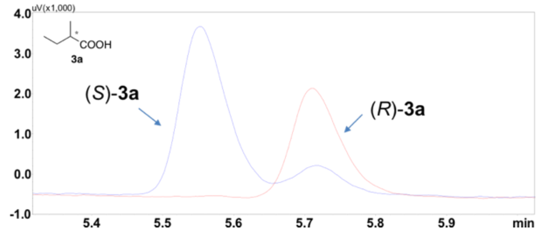

uV(x1,000)
4.0
3.0
2.0
1.0
0.0
-1.0
COOH
3a
(S)-3a
(R)-3a
5.4
5.5
5.6
5.7
5.8
5.9
min

3b

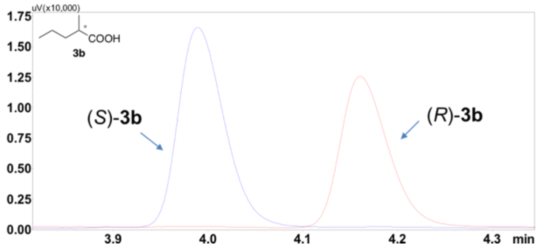

uV(x10,000)
1.75
1.50
1.25
1.00
0.75
0.50
0.25
0.00
COOH
3b
(S)-3b
(R)-3b
3.9
4.0
4.1
4.2
4.3
min

3c

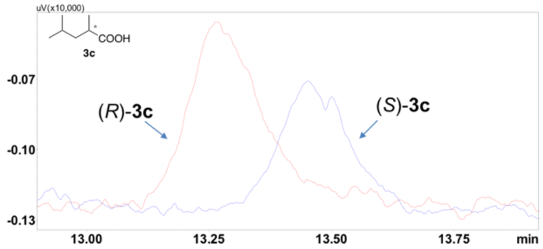

uV(x10,000)
-0.07
-0.10
-0.13
COOH
3c
(R)-3c
(S)-3c
13.00
13.25
13.50
13.75
min

3d

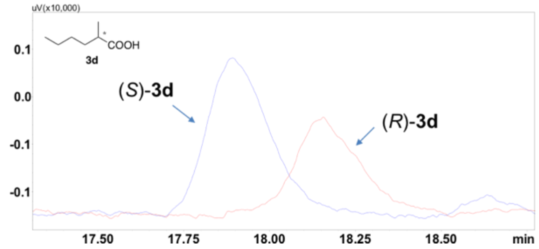

uV(x10,000)
0.1
0.0
-0.1
-0.1
COOH
3d
(S)-3d
(R)-3d
17.50
17.75
18.00
18.25
18.50
min

References

(1) Okrasa, K.; Levy, C.; Wilding, M.; Goodall, M.; Baudendistel, N.; Hauer, B.; Leys, D.; Micklefield, J. Structure-Guided Directed Evolution of Alkenyl and Arylmalonate Decarboxylases. *Angew. Chem. Int. Ed.* **2009**, *48* (41), 7691–7694.

(2) Miyamoto, K.; Ohta, H. Asymmetric Decarboxylation of Disubstituted Malonic Acid by Alcaligenes Bronchisepticus KU 1201. *Biocatalysis* **1991**, *5* (1), 49–60.
